# Polarized retinal pigment epithelium generates electrical signals that diminish with age and regulate retinal pathology

**DOI:** 10.1111/jcmm.13829

**Published:** 2018-08-30

**Authors:** Lin Cao, Jie Liu, Jin Pu, Gillian Milne, Mei Chen, Heping Xu, Alan Shipley, John V Forrester, Colin D McCaig, Noemi Lois

**Affiliations:** ^1^ School of Medicine Medical Sciences and Nutrition Institute of Medical Sciences University of Aberdeen Aberdeen UK; ^2^ Yizhou International Proton Medical Centre and Cancer Hospital He Bei China; ^3^ Department of Ophthalmology Frist Hospital Affiliated to the Chinese PLA General Hospital Beijing China; ^4^ Wellcome‐Wolfson Institute for Experimental Medicine Queen's University Belfast UK; ^5^ Biological Research & Development University of New England Biddeford Maine

**Keywords:** ATP1B1, CCL2/CX3CR1 double knockout mice, cell‐cell connection, extracellular electrical signalling

## Abstract

The transepithelial potential difference (TEP) across the retinal pigment epithelial (RPE) is dependent on ionic pumps and tight junction “seals” between epithelial cells. RPE cells release neurotrophic growth factors such as pigment epithelial derived factor (PEDF), which is reduced in age‐related macular degeneration (AMD). The mechanisms that control the secretion of PEDF from RPE cells are not well understood. Using the CCL2/CX3CR1 double knockout mouse model (DKO), which demonstrates RPE damage and retinal degeneration, we uncovered an interaction between PEDF and the TEP which is likely to play an important role in retinal ageing and in the pathogenesis of AMD. We found that: (a) the expression of ATP1B1 (the Na^+^/K^+^‐ATPase β1 subunit) was reduced significantly in RPE from aged mice, in patients with CNV (Choroidal Neovascularization) and in DKO mice; (b) the expression of PEDF also was decreased in aged persons and in DKO mice; (c) the TEP across RPE was reduced markedly in RPE cells from DKO mice and (d) an applied electric field (EF) of 50‐100 mV/mm, used to mimic the natural TEP, increased the expression and secretion of PEDF in primary RPE cells. In conclusion, the TEP across the RPE depends on the expression of ATP1B1 and this regulates the secretion of PEDF by RPE cells and so may regulate the onset of retinal disease. Increasing the expression of PEDF using an applied EF to replenish a disease or age‐reduced TEP may offer a new way of preventing or reversing retinal dysfunction.

## 1 BACKGROUND

The retinal pigment epithelium (RPE) is a polarized epithelial monolayer which lies between the photoreceptor cells of the retina and the choriocapillaris layer of the choroid. The RPE has many functions, for example light absorption, trans‐epithelial transport, phagocytosis, secretion of growth factors and protection of the neural retina. Dysfunction and death of RPE cells play critical roles in the pathogenesis of several retinal disorders.^1^, ^2^ For example, RPE dysfunction occurs very early on in diabetic retinopathy, even before visual loss or overt diabetic retinopathy is evident clinically.^3^ One feature of healthy RPE is the generation of a trans‐epithelial potential difference (TEP) of around 3.5 mV (apical side positive).^4^ Because the RPE has an average thickness of about ~50 μm, the voltage gradient across the RPE (TEP) gives rise to a physiological electrical field (EF) of ~70 mV/mm. The TEP is created by the establishment of ionic gradients across the RPE. These are driven for example by membrane transporters such as the polarized Na^+^/K^+^‐ATPase pumps and maintained by tight junctions between epithelial cells. These have a high trans‐epithelial electrical resistance (TEER) of 448 Ω/cm^2^ that prevents ionic back flux and so preserves the ionic gradients.^5^

The functional role of the TEP across the RPE is not fully understood, but in other tissues endogenous electrical signals regulate a variety of cellular and molecular functions.^6^, ^7^, ^8^, ^9^ Direct measurements have shown that an extracellular electrical signal of 42 mV/mm is present at a bovine corneal wound^10^ and that similar or smaller applied electric fields (EFs) directed migration of corneal epithelial cells (CECs)^11^, ^12^ and RPE cells.^13^ In addition, division of CECs is oriented by a small applied EF.^14^ The TEP also influences the transport of fluid/substances across the RPE, as it does for instance, in the kidney and urothelium, where the TEP contributes to tubular reabsorption and to ATP release.^15^ We hypothesize therefore that the TEP may play a functional role in maintaining the normal health of the retina.

Pigment epithelium‐derived factor (PEDF) is a naturally occurring glycoprotein secreted from the apical side of RPE cells.^16^, ^17^, ^18^ It has broad bioactive properties for the health of normal retina, including neuroprotective,^19^, ^20^, ^21^ antiangiogenic 22, 23, 24 and anti‐senescent functions.^25^, ^26^ Local expression of PEDF is decreased significantly in the RPE of patients with AMD, and this is responsible in part for the pathogenesis of the disorder.^27^

The double knockout mice CCL2/CX3CR1 without rd8 mutation (DKO mice) which we used here have deletions of the chemokine CCL2 and receptor CX3CR1 which predispose mice to age‐ and light‐mediated RPE and retinal damage, but does not include pathogenic retinal angiogenesis.^28^, ^29^, ^30^, ^31^, ^32^ In addition, Ccl2‐knockout (Ccl2^−/−^) mice develop drusen‐like changes, accumulations of extracellular material between Bruch's membrane and the RPE and also RPE atrophy.^33^, ^34^ RPE degeneration occurs by 9 months in Ccl2‐deficient mice^33^ and a significant decrease in PEDF expression in mouse retina and RPE was found in vivo and in cultured DKO RPE cells.^35^ We sought therefore to determine the interactions between TEP and PEDF secretion and also the functional roles of each signal on healthy and diseased RPE.

## 2 MATERIALS AND METHODS

### 2.1 Animals

Three to fifteen months‐old DKO mice (n = 18, 6 at 3‐4 months, 4 at 6 months, 4 at 9 month and 4 at 15 months) and age‐matched C57BL/6J wild‐type (WT) control mice were used. CCL2/CX3CR1 DKO mice were kindly supplied by Prof. Xu (Queens University, Belfast). The DKO mice do not carry Crb1 rd8 mutation.^32^ DKO mice were maintained in a standard animal housing room with a 12‐hour light/dark cycle in the Biological Research Unit (BRU) at Queen's University Belfast. WT mice were obtained from the Medical Research Facility, University of Aberdeen. All in vivo procedures were undertaken under the regulation of UK Home Office Animals (Scientific Research) Act 1986. The study was conducted in compliance with the Association for Research in Vision & Ophthalmology Statement for the Use of Animals in Ophthalmology and Vision Research.

### 2.2 RPE cell isolation and culture

RPE cells were isolated and cultured from WT, DKO mice eyes and normal person's eyes as described previously.^36^, ^37^, ^38^ Using eyes provided by the Bristol and Manchester Eye Banks and after the cornea was removed for the purpose of transplantation, RPE cells were gathered and 9 human RPE cell lines, 3 from persons less than 50 years old (“young”) and 6 from over 70 year old persons (“old”), established. In brief, after removal of the anterior segment of the eye and the lens, the neuronal retinas were peeled off from the eyecups under the dissecting microscope. The RPE/choroid/sclera cups were filled with 0.5% (w/v) trypsin‐EDTA (ICN Flow, Irvine, UK) and incubated at 37°C for 1 hour. For mice, the eye was incubated for digestion after cornea and lens removal. Then, the RPE was peeled off from the choroid under microscope for cell culture and Western blotting. The RPE cells were released from the basement membrane by gentle aspiration. After two washes, single cell suspensions were cultured in a 35‐mm dish with Dulbecco's modified Eagle's medium (DMEM, Invitrogen) containing 10% (v/v) foetal calf serum (FCS, Sigma). The first passage was used for protein expression assays using Western blot. Second and third passage cells were used for measurement of trans‐epithelial electrical resistance (TEER), trans‐epithelial potential difference (TEP) and other experiments.

### 2.3 TEP detections using Millicell‐ERS system

1‐2 × 10^5^ primary cultured RPE cells from wild type and DKO mice were seeded on 24‐well cell culture inserts to form monolayers (Millipore). The inserts contain a 0.4‐μm pore size polycarbonate membrane pre‐coated with collagen type I. The medium was replaced every 48 hours. TEER and TEP were determined using a Millicell ERS‐2 Voltmeter (MERS00002, EMD Millipore) at 1‐4 weeks.

### 2.4 Measuring the electrical current on the RPE

The scanning vibrating electrode technique (SVET, Applicable Electronics) was used to determine the endogenous electrical current of the RPE.^39^ The probe vibration is controlled by a piezoceramic displacement device allowing vibration amplitudes from 1 to 30 μm (perpendicular to the sample surface). Every 35‐μm one measurement point of the vertical component of the current density was recorded to build up the entire current density map. The RPE cells were seeded in a 35‐mm dish to form a monolayer after 21 days culture. All system parameters, including the xyz scanning mechanism, piezo actuator and lock‐in amplifier, are controlled via a PC using ASET software from Science Wares Inc., USA.

### 2.5 Immunofluorescent staining and imaging

Cells were fixed in 4% paraformaldehyde for 20 minutes, followed by permeabilization (5 minutes) and blocking (30 minutes). The cells were stained for 2 hours with antibodies to Na^+^/K^+^‐ATPase (α1 and β1 subunits, EMD Millipore), E‐cad (BD Biosciences) and ZO‐1 (Invitrogen, UK), respectively, and then were incubated with secondary antibodies (Invitrogen) and phalloidin‐TRITC (Sigma‐Aldrich, UK) for 1 hour. Images were obtained with the Zeiss Axio Observer Z1 inverted fluorescence microscope and Confocal Zeiss 710 LSM (Carl Zeiss, Germany).

### 2.6 Immunohistochemistry staining

Mouse eyes were fixed with 2% paraformaldehyde for 2 hours. After paraffin embedding, the eyeballs were cut into 5‐μm‐thick sections and mounted on charged glass slides. Slides were de‐paraffinized and subjected to citrate‐based antigen retrieval. Paraffin sections were re‐treated with the DAKO high pH antigen retrieval system (DAKO, Carpinteria, CA) using a domestic 600 kW microwave oven. Nonspecific antibody binding was blocked by incubating sections in 4% BSA followed by 10% non‐immune goat serum (Zymed Corp., San Francisco, CA). Primary antibody was applied at a 1:200 to 400 dilutions overnight at room temperature. Sections then were incubated with secondary antibody for 30 minutes. The localization of target proteins was demonstrated with pre‐diluted streptavidin‐horseradish peroxidase (Zymed) and 0.05% 3, 3‐diaminobenzidine in TBS, with H_2_O_2_ as the substrate. All sections were counterstained lightly with haematoxylin.

### 2.7 Western blot

Western blot (WB) was performed as described previously.^40^ Primary antibodies used included anti‐ATP1A1 (Abcam), ATP1B1 (EMD Millipore), E‐cadherin (BD Biosciences), ZO‐1 (Invitrogen, UK), PEDF and GAPDH (Santa Cruz, USA). The immunoblots were detected by Clarity Western ECL Substrate (Bio‐Rad). Cell lysates were collected using RIPA buffer for further WB experiments.

### 2.8 Applied electrical stimulation in vitro

DC electric fields were applied to primary cultured RPE cells in electrotactic chambers as described before^41^ (Figure 6A). A DC electric field of 50‐100 mV/mm was applied and measured directly using an ammeter (34410A digital multimetre, Agilent Technologies). The samples were exposed to an applied EF for 1 and 3 hours, and then, cell pellets were prepared for protein assays.

### 2.9 Detection of neurotrophic factors secretion from RPE by ELISA

The conditioned medium was collected from different side of cultured RPE cells in a transwell cell culture system (Mlillicell UK). The concentration of PEDF, HGF and BDNF was determined by ELISA using commercial kits (RayBiotech UK). ELISA was performed using the manufacturer's instructions.

### 2.10 Microarray data analysis

The microarray data sources were from the Gene Expression Omnibus (GEO).^42^ Two data sets (series accession number of GSE29801 and GSE10965) were not subjected to any additional normalization, as all had been normalized when these were obtained. Using the GSE10965 data set, the gene expression of retinal pigmented epithelium/choroid from young and old animals was compared, including 4 samples from young mice and 4 samples from old mice. Each sample contained 4 retinal pigmented epithelium/choroid from 2 animals.^43^ Using GSE29801, a systems‐level transcriptome analysis of the retina and retinal pigment epithelium (RPE)‐choroid complex from 31 normal, 26 AMD and 11 potential pre‐AMD human eyes was performed using Agilent‐014850 Whole Human Genome Microarray.^44^ We analysed the expression of ATP1B1 and PEDF in these published microarray data sets on line. The identity of genes across microarray data sets was established using public annotations, primarily based on Unigene.^45^

### 2.11 Statistical analysis

A minimum of three replicates was undertaken and analysed for each experiment presented. Data are shown as the mean ± SEM. Student's *t* test was used to test for significant differences between groups. Differences were considered statistically significant if the *P* value was <0.05.

## 3 RESULTS

### 3.1 Reduced ATP1B1 expression in RPE of aged mice and retina from patients with AMD

**Figure 1:**
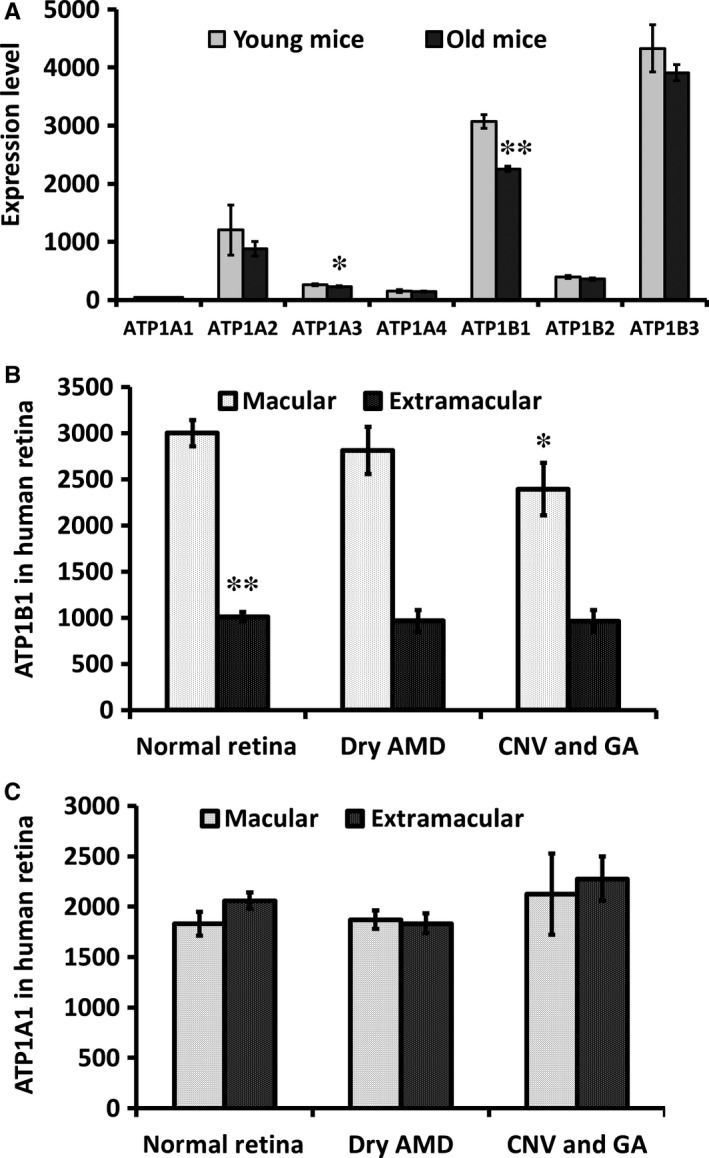
The expression of ATP1B1 was down‐regulated in aged RPE and wet AMD. A, We analysed the data set on the GEO (gene expression omnibus, NCBI). The expression of ATP1B1 was reduced significantly in aged mice (4 vs 26 months, *P* < 0.01). B, the expression of ATP1B1 and ATP1A1 in normal and AMD retina were analysed. ATP1B1 expression in normal macula (n = 28) was three times higher than from extramacular areas (n = 27, *P* < 0.01). ATP1B1 expression in both macula with CNV (Choroidal Neovascularization) and with GA (geographic atrophy) (n = 9) was reduced significantly compared to that in normal macula (n = 28, *P* < 0.05). C, Expression of ATP1A1 was not different between macula and extramacular or normal and AMD. **P < 0.01, *P < 0.05

We analysed the microarray data on Gene Expression Omnibus (GSE29801 and GSE10965).^44^ In these group microarray data, we analysed expression of ATP1B1 and ATP1A1 in RPE of young and old mice and 175 samples from the macular or extramacular region of human donor eye RPE‐choroids and 118 samples from the macular or extramacular region of human donor retina with no reported ocular disease, possible preclinical AMD or AMD. The results showed that ATP1B1 was down‐regulated significantly in old mice (Figure 1A). In sample analysis from patients with AMD, there was much higher expression of ATP1B1 in macula of human retina (*P* < 0.001), but no significant difference in expression of ATP1A1 between macula and extramacula retina (Figure 1B and C). Importantly, we found that the expression of ATP1B1 was reduced significantly in macula of AMD with CNV (Choroidal Neovascularization) and GA (geographic atrophy) (*P* < 0.05). Our analysis suggested strongly that the decreased expression of ATP1B1 could be a specific factor which may correlate with wet AMD and GA.

### 3.2 Secretion of PEDF and other neurotrophic factors was reduced in cultured RPE

**Figure 2:**
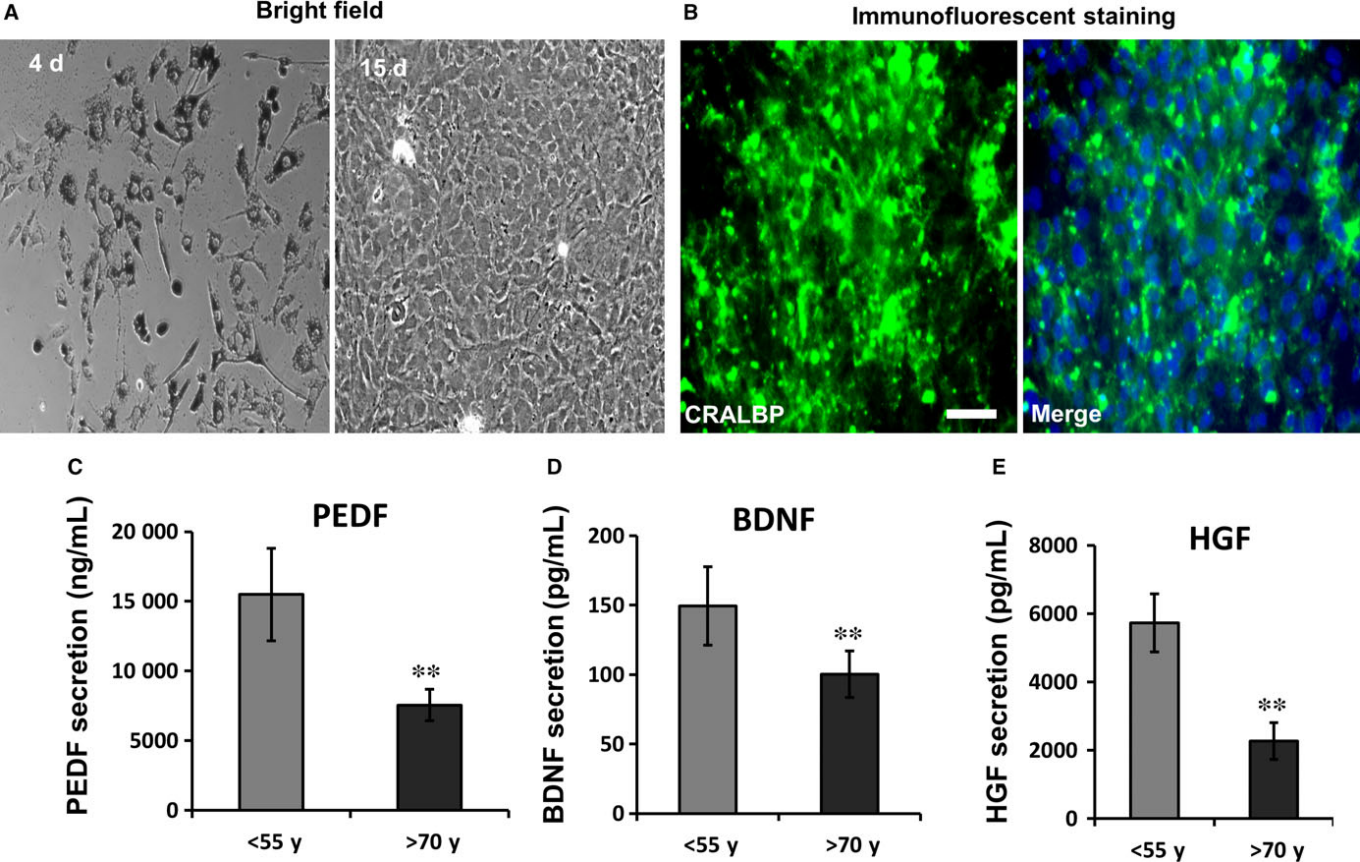
Reduced secretion of neurotrophic factors in aged primary cultured RPE cells. A, under bright field, cultured RPE cells had richly pigmented cytoplasm at 4 d. By passage 3, RPE cells had lost their pigment after 15‐day culture. B, CRALBP is a specific marker of RPE. It is significantly expressed in passage 3 primary cultured RPE (immunofluorescent staining). Green is CRALBP and Blue is DAPI. C‐E, In cultured RPE cells from different aged persons, the secretion of PEDF, BDNF and HGF all were reduced significantly in the “old” group (>70 year old; n = 6) compared with the younger group (<55 year old; n = 3). *P < 0.05, **P < 0.01. Bar in A and B is 50 μm

Next we checked the secretion of PEDF in cultured RPE cells from people of different ages. We confirmed the RPE identity of our cells from their expression of the RPE‐specific marker CRALBP (Cellular Retinaldehyde‐binding Protein, Figure 2A and B). After 2‐week culture, we harvested the culture medium and assessed the concentrations of PEDF, BNDF and HGF from “young” and “old” patients (see Methods: Figure 2C‐E). We found that PEDF was reduced in the “old” age group from 15 489 ± 230 ng/mL to 7560 ± 180 ng/mL, BDNF from 145.9 ± 62 pg/mL to 100.2 ± 26 pg/mL and HGF from 5727 ± 1100 pg/mL to 2268.2 ± 230 pg/mL (all statistically significant, *P* < 0.01). These data indicate that the expression of ATP1B1 (Figure 1A) and secretion of PEDF in retina fall off with increasing age (Figure 2C‐E).

### 3.3 Reduced ATP1B1 and PEDF expression in RPE and retina in DKO mice

**Figure 3:**
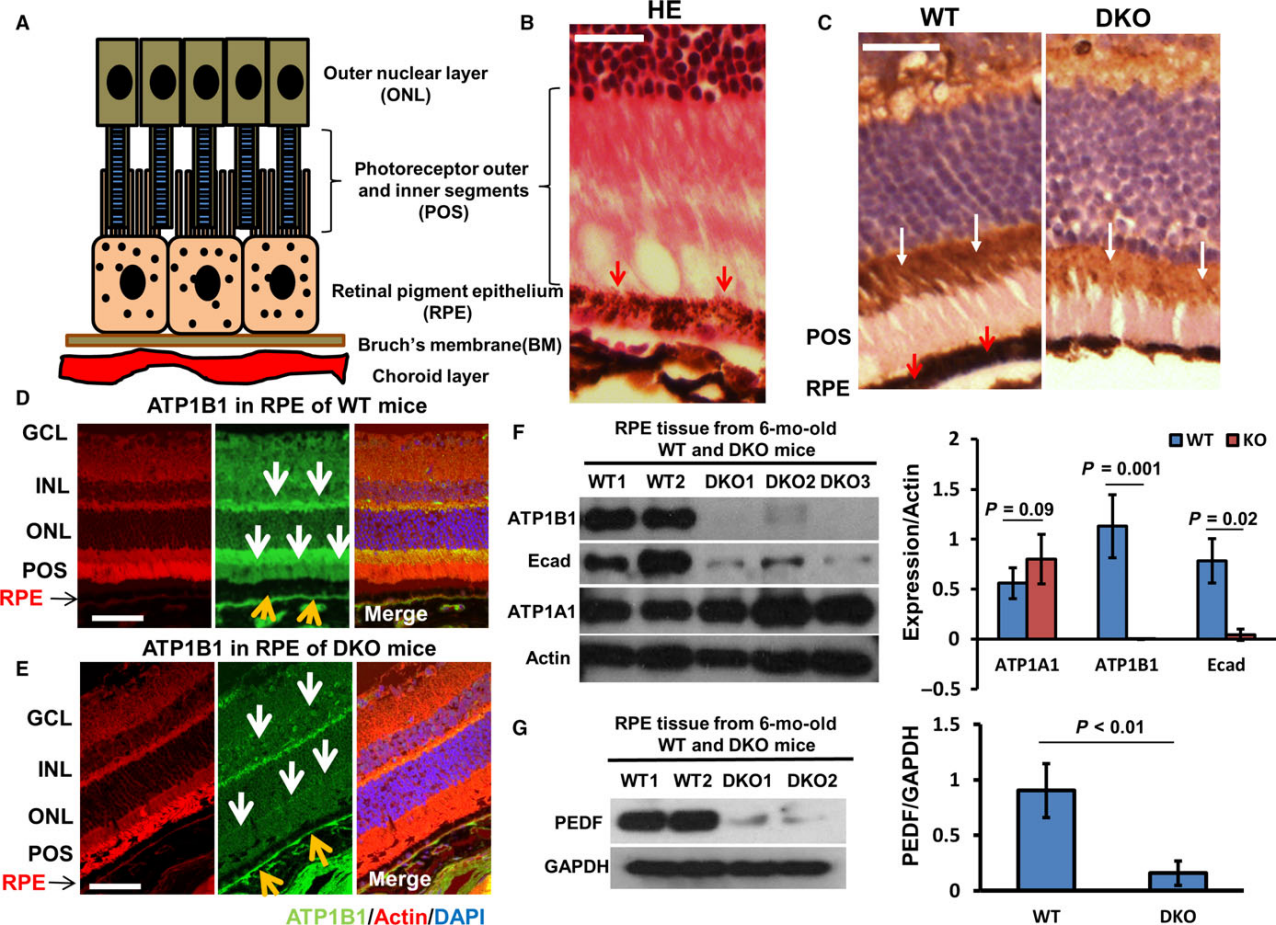
Down‐regulation of ATP1B1, E‐cad and PEDF expression in RPE of DKO mice. A, Schematic diagram of RPE and associated structures. B, RPE, photoreceptor outer and inner segments (POS) and outer nuclear layer (ONL) stained with haematoxylin and eosin (HE), and the pigment particles in RPE are outlined by red arrows. C, Left panel, in RPE (red arrows) and POS, the ATP1B1 was stained by IHC (immunohistochemistry) to show the black particles in RPE and POS (white arrows). Right panel, IF (immunofluorescent staining) showed that the reduced ATP1B1 (highlighted by white arrow) located at the POS in DKO mice. D, In WT, the expression of ATP1B1 was presented by IF staining (white and yellow arrow). E, In DKO mice, ATP1B1 expression was reduced significantly on the apical side of RPE, POS and GCL (yellow and white arrows). F, Western blots showed that the expression of ATP1B1 and E‐cad was reduced markedly, but that ATP1A1 expression was not reduced in RPE of 6‐mo‐old DKO mice. Actin is loading control. G, Western blots showed that the expression of PEDF was down‐regulated in RPE of 6‐mo‐old DKO mice. GAPDH is loading control. The histograms in F and G show the relative intensities of ATP1A1, ATP1B1, E‐cad, PEDF expressed as a ratio with respect to the loading control (n = 4 in WT and DKO each) GCL, ganglion cell layer; INL, inner nuclear layer; IPL, inner plexiform layer; INL, inner nuclear layer; ONL, outer nuclear layer; POS, photoreceptor outer and inner segments. Bar is 45 μm

DKO mice are a well‐recognized model of retinal dysfunction diseases with RPE degeneration. We compared the expression of ATP1A1 (Na^+^/K^+^‐ATPase alpha1), ATP1B1 (Na^+^/K^+^‐ATPase beta1), E‐Cad (adhesion junction, E‐cadherin) and PEDF in RPE from DKO and age‐matched WT mice. In WT mice, ATP1B1 was located more on the apical side (including the photoreceptor layer) than on the basal side of RPE and was distributed widely in different layers of the retina (Figure 3A‐D). In DKO mice, the expression of ATP1B1 on the apical side of RPE and on the photoreceptor outer segment layer was reduced significantly by 6 months (Figure 3C and E). Expression of ATP1B1, E‐Cad and PEDF also was reduced significantly in 6 month DKO RPE (western blots, Figure 3F and G). ATP1A1 expression, however, was unchanged in DKO mice (Figure 3F). Formation of tight cell‐cell connections (functions of E‐Cad and ZO‐1) and apical localization of the subunits of Na^+^/K^+^‐ATPase on the RPE are essential to generate the electrical signal that is the TEP. Collectively, these data indicate that the TEP may be reduced with AMD, because it depends on the ageing epithelium maintaining Na^+^/K^+^‐ATPase and tight junction (TJ) functions in RPE.

### 3.4 Age and AMD reduce ATP1B1, ZO‐1 and the TEP in RPE

**Figure 4:**
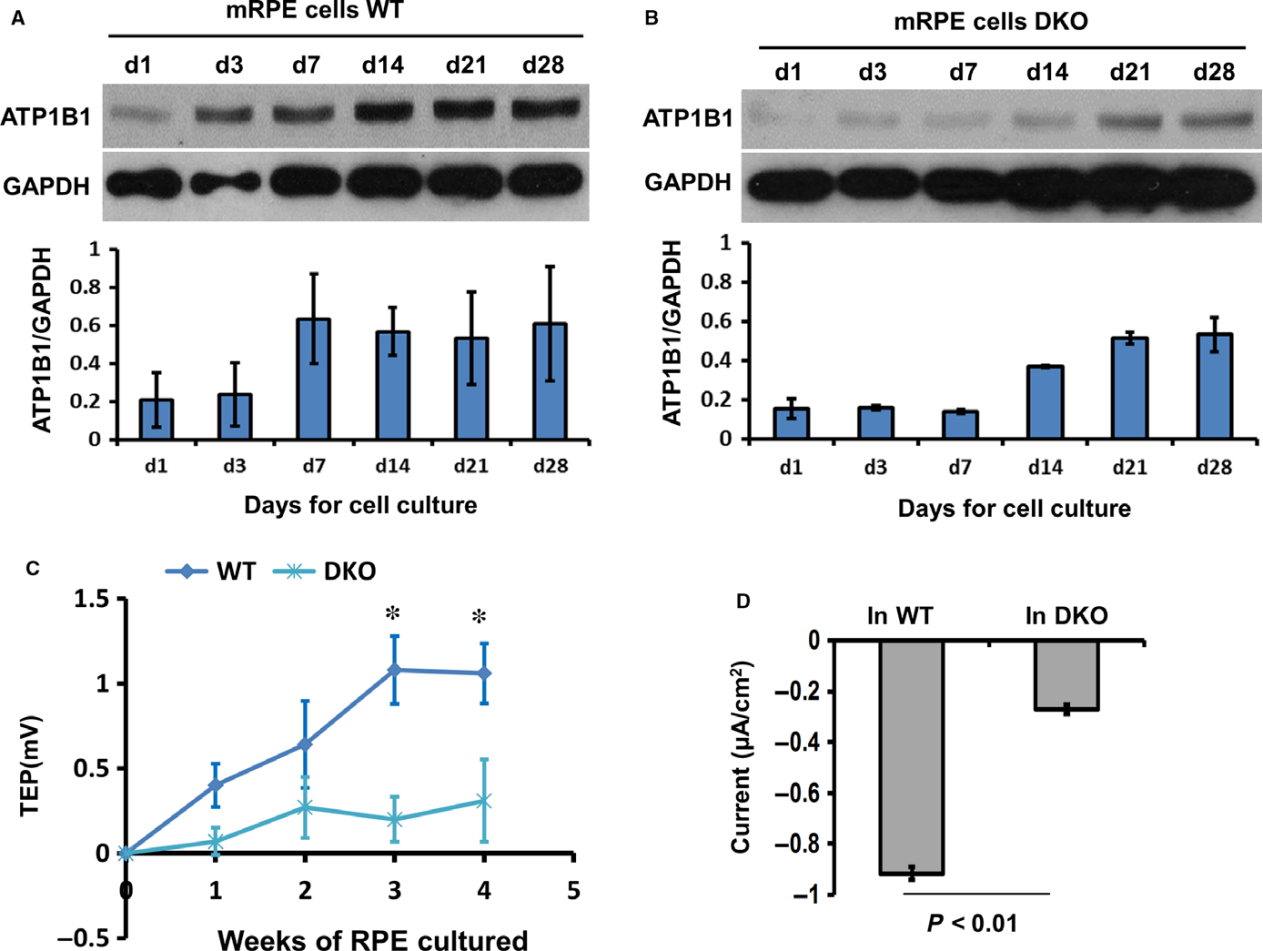
DKO RPE had reduced TEP and reduced expression of ATP1B1. A and B, Western blots show ATP1B1 expression was up‐regulated in WT RPE cells in 3 d. In DKO mice, the up‐regulation of ATP1B1 was delayed by at least 1 wk appearing only after 14 days in culture. GAPDH is loading control. The histograms in A and B show the relative intensity of ATP1B1 expressed as a ratio with respect to the loading control. C, Using transwell culture and Millicell ERS, the trans‐RPE electrical potential difference (TEP) was determined. At 3 wk in culture, the TEP was fourfold less in DKO RPE compared with WT (1.1 ± 0.2 mV WT and 0.25 ± 0.2 mV DKO,* P* < 0.05). D, After 3 wk in culture of RPE monolayers, electrical current was detected using SVET (Scanning vibrating electrode technology) which detects current flowing through the entire epithelial surface it scans. The average of electrical current was threefold greater in WT than in DKO mice (−0.9 ± 0.12 μA/cm^2^ and −0.28 ± 0.2 μA/cm^2^, respectively, d3). All results were from three or more independent experiments

**Figure 5:**
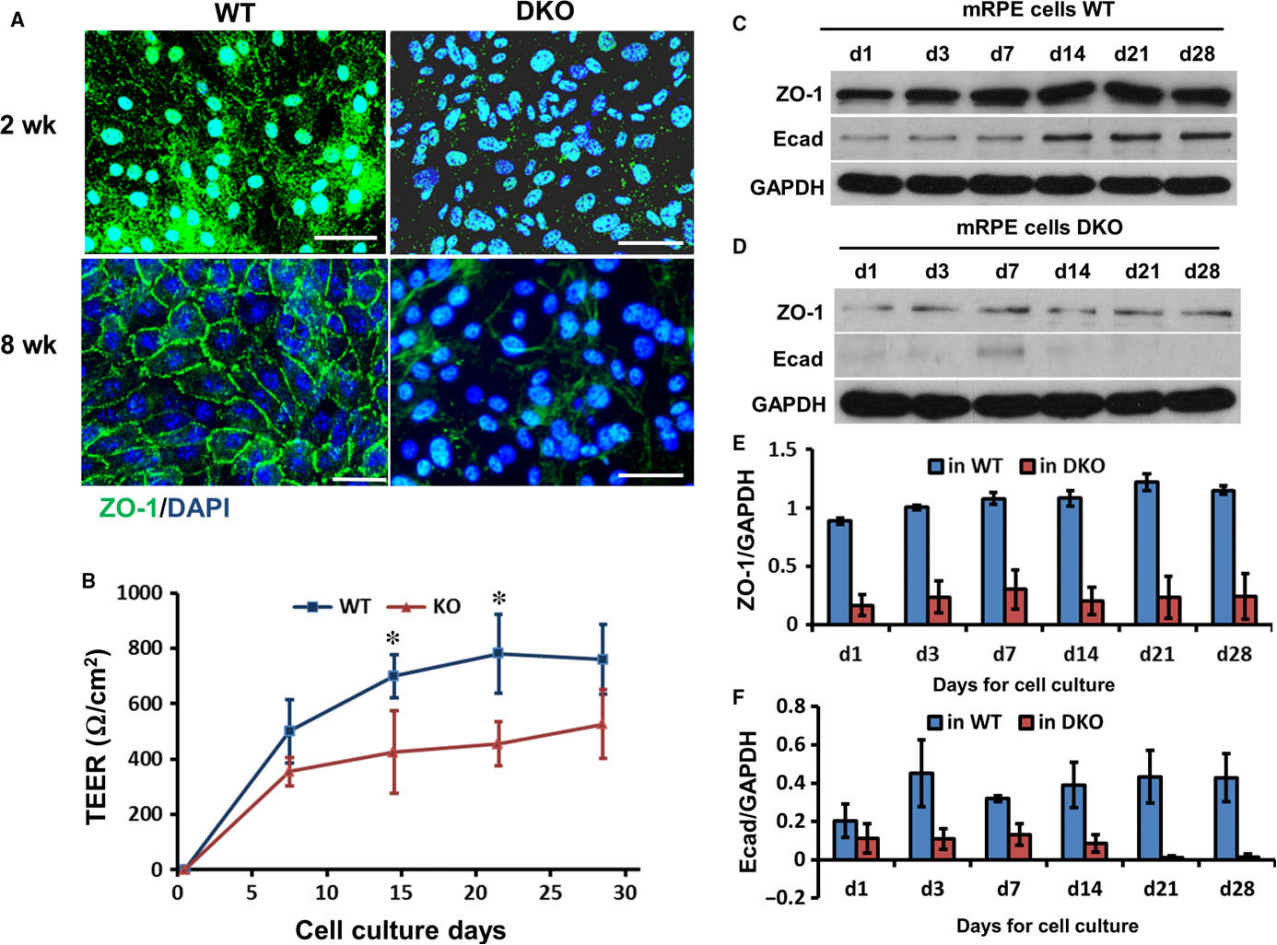
In DKO mice tight junctions in the RPE were disrupted. A, ZO‐1 was expressed on the membrane of primary cultured RPE monolayers to delineate clearly junctional borders between cells from WT mice after 8‐wk culture. In DKO mice, ZO‐1 expression was reduced on the membrane of cells and cell‐cell boundaries were ill‐defined or missing (see lower right panel), N = 3. B, Transepithelial electrical resistance (TEER) was determined in RPE monolayers in WT and DKO mice using Millicell‐ERS. TEER was inhibited by close to 50% in DKO mice at 2 and 3 wk of culture, N = 6, *P* < 0.05. C‐F, The protein lysate of primary cultured RPE cells was harvested at different time‐points and analysed by Western blots. ZO‐1 expression increased in 3 d and E‐cad increased in 14 d in WT mice. In DKO mice, expression of ZO‐1 and E‐cad were not up‐regulated significantly during 28‐d culture. Histogram shows the relative band intensities of E‐cad and ZO‐1 measured by ImageJ and normalized as a ratio with GAPDH. GAPDH was a loading control, N = 3. Bar is 20 μm

Adult human RPE has an apically positive TEP of 3.5 mV.^4^ The TEP is an inherent property of transporting epithelia and arises from spatial variations in ion pumps, channels and leak conductances across layers of cells.^5^ The Na^+^/K^+^‐ATPase and cell‐cell tight junctions are important in the generation and maintenance, respectively, of the TEP and incorrect localization of Na^+^/K^+^‐ATPase can cause disease, for example autosomal dominant polycystic kidney disease.^46^, ^47^ We found ATP1B1 expression was reduced in aged mice and mice with AMD. Perhaps retinal disease is also associated with a reduction in TEP. In cultured epithelial cells, the expression of Na^+^/K^+^‐ATPase increases with time as the polarity develops.^48^ We found that the expression of ATP1B1 was up‐regulated in WT mouse RPE cells (3 days in culture; Figure 4A), but that expression levels of ATP1B1 and ZO‐1 were reduced significantly in DKO RPE and became up‐regulated only much later, after 14 days in culture (Figures 4B and 5A, C and D). In transwell cultures, RPE monolayers polarize and develop a trans‐epithelial potential difference (TEP) and a transepithelial electrical resistance (TEER).^49^ Here, the TEP and TEER were measured using a Millicell ERS‐2 Voltammeter and a transwell culture system. We found that the TEP across DKO RPE were as much as fourfold less than that from WT mice in 3‐week cultures (0.25 ± 0.2 mV DKO, compared to 1.1 ± 0.2 mV, positive apically, *P* < 0.05; Figure 4C) and the TEER was reduced significantly in RPE from DKO mice (Figure 5B). According to Ohm's law (I = V/R), we calculated the electric current flowing across 2 week cultured RPE and found that the I_DKO_ was 0.93 ± 0.21 μA/cm^2^ nearly 30% less than the I_wt_ which was 1.19 ± 0.13 μA/cm^2^ (apical positive, with current directed inward). To confirm our data, we determined the change of electrical current using scanning vibrating electrode technology (SVET) which sensitively maps current flow on the surface of primary cultured RPE cells. We found an inward electrical current (negative value) that was fourfold lower in RPE of DKO mice than in WT mice (after 2 weeks in culture; −0.21 ± 0.1 μA/cm^2^ compared with −0.92 ± 0.1 μA/cm^2^, *P* < 0.01) (Figure 4D). These data confirm that the TEP was reduced significantly in mice with a deficiency of CCL2/CX3CR1, most probably because of the down‐regulation of Na^+^/K^+^‐ATPase and the defective TJs.

### 3.5 Reduced levels of PEDF and BNDF in DKO mice are rescued by an applied EF

**Figure 6:**
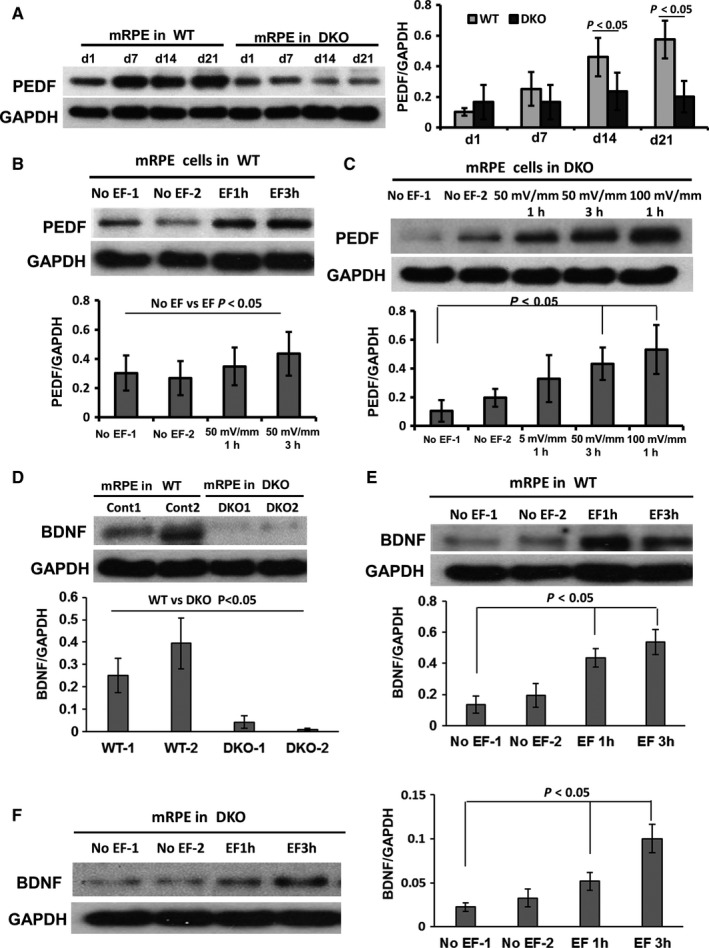
An applied EF of similar strength to the TEP increased expression of PEDF and BNDF in RPE. A, The expression of PEDF in primary cultured RPE from WT and DKO mice was determined by Western blot. In WT mice, the expression of PEDF increased over 1‐3 wk in culture. However, in DKO mice the expression of PEDF did not increase over 3 wk. B and C, An applied EF of 50‐100 mV/mm (similar in strength to the TEP) increased the expression of PEDF in 2 d of cultured RPE from both WT and DKO mice. D‐F BDNF showed low expression in DKO RPE and this was increased by an applied EF of 50 mV/mm in WT and DKO RPE. The histograms in A to F show the relative intensity of PEDF and BDNF expressed as a ratio with respect to the loading control. GAPDH is loading control. All the results were from three independent experiments

We showed above that the expression of ATP1B1, E‐Cad, PEDF and TEP all were reduced significantly in 6 month DKO RPE. These data indicate again that the TEP (based on Na^+^/K^+^‐ATPase and cell‐cell connections) and PEDF may be linked in retinal degenerative disease. Here, we used an applied EF, similar in strength to the endogenous TEP, to stimulate the RPE and determine the correlation between TEP and PEDF in vitro. First, we showed that PEDF and BNDF expression increased significantly in WT RPE over time in culture during the process of monolayer polarization, but that this did not happen in DKO mice (7 days culture; Figure 6A and D). Then, we showed that an applied EF of 50‐100 mV/mm (equivalent to the TEP), markedly up‐regulated PEDF and BNDF expression (in a voltage‐dependent manner) in both WT and DKO RPE (Figure 6B, C, E and F), indicating that the TEP across RPE regulates PEDF expression and controls the PEDF secretion from RPE. Importantly, an applied EF restored the reduced PEDF levels in DKO mice to normal. This suggests that a reduced TEP may contribute to the retinal degeneration which arises from a reduction in PEDF expression in RPE. Furthermore, our data suggest that EF‐induced secretion of PEDF may represent a new therapeutic means of treating retinal disease.

### 3.6 An applied electric field increased PEDF secretion

**Figure 7:**
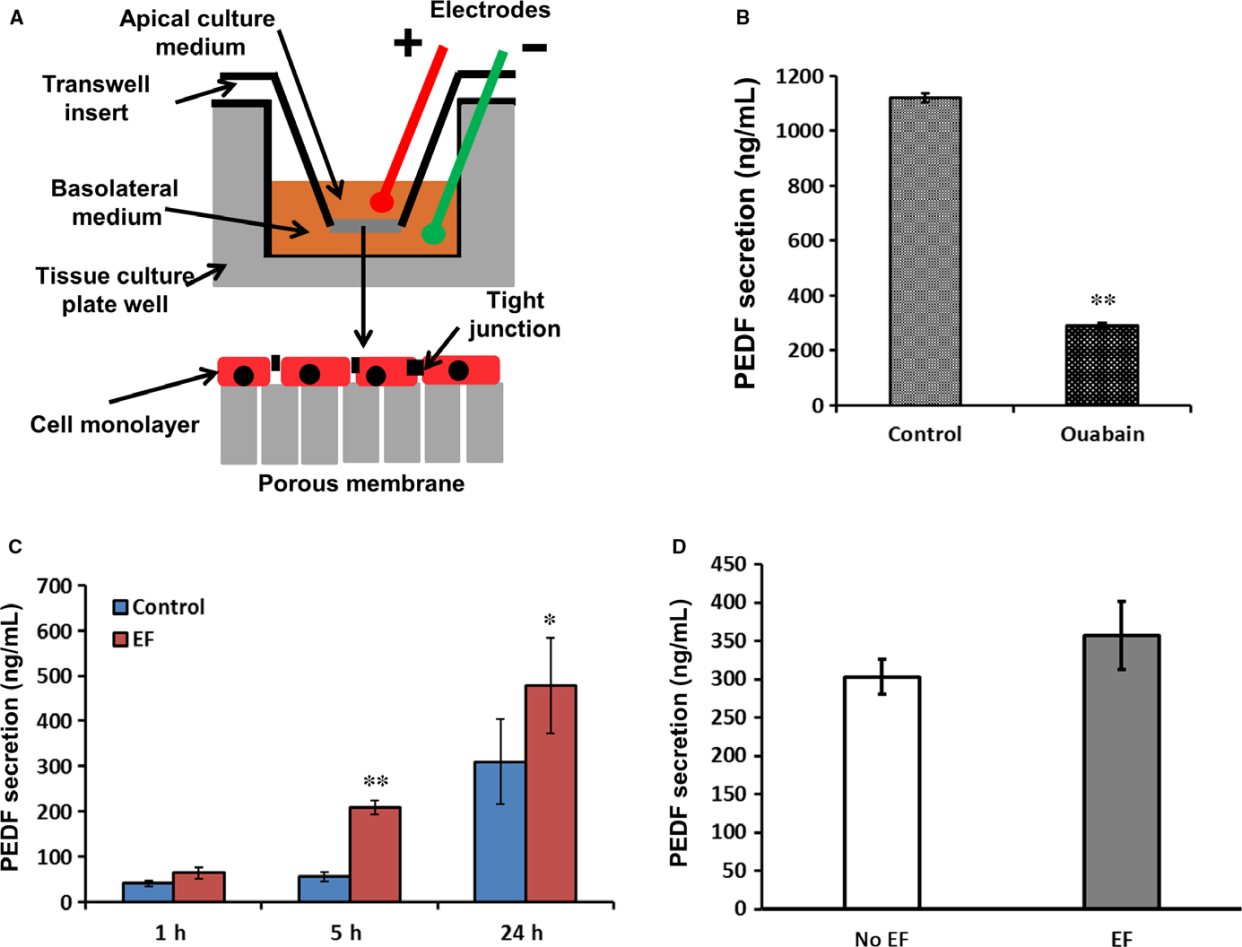
An applied EF increased the secretion of PEDF from RPE monolayers. A, Schematic diagram of electrical stimulation in a transwell plate. B, After 2‐week culture in transwell plate, the renewed medium on the apical side was harvested from control monolayers and those treated with ouabain for 24 h. The concentration of PEDF in the apical medium of untreated monolayers was 1180 ± 36 ng/mL; from those treated with ouabain PEDF concentration was almost fourfold less; 305 ± 28 ng/mL (*P* < 0.01). C. When we stimulated cells with a DC EF, anode apically, the secretion of PEDF significantly increased after 5 h treatment and this was maintained at 24 h. D, When cells were exposed to the same strength of EF but with the cathode apically (a non‐physiological polarity), the secretion of PEDF did not increase compared to untreated monolayers. The strength of applied EF was 100 mV/mm. *P < 0.05, **P < 0.01

Finally we determined whether the applied EF regulated the secretion of PEDF from RPE (Figure 7A). Firstly, we checked the secretion of PEDF into the culture medium with/without ouabain which is an inhibitor of the TEP. We found that ouabain reduced the secretion of PEDF almost fourfold in transwell cultures of RPE (Figure 7B). Next we stimulated the RPE cells by applying an EF with the anode at the apical side, the normal physiological polarity and with a field strength of 100 mV/mm (similar to the TEP; Figure 7C). After 5 hours, PEDF secretion had increased about fourfold (from 55.9 ± 12 ng/mL to 200.8 ± 14 ng/mL; Figure 7C) and was still elevated by about 50% after 24‐hour EF stimulation (from 310.2 ± 110 ng/mL to 478.2 ± 115 ng/mL; Figure 7C). In striking contrast, PEDF secretion remained unchanged with an EF of the same physiological strength, but with the cathode at the apical side, the reverse of the normal physiological polarity (Figure 7D). These data show that applying an EF of both physiological magnitude and polarity to mimic the TEP across the RPE regulated the secretion of PEDF.

**Figure 8:**
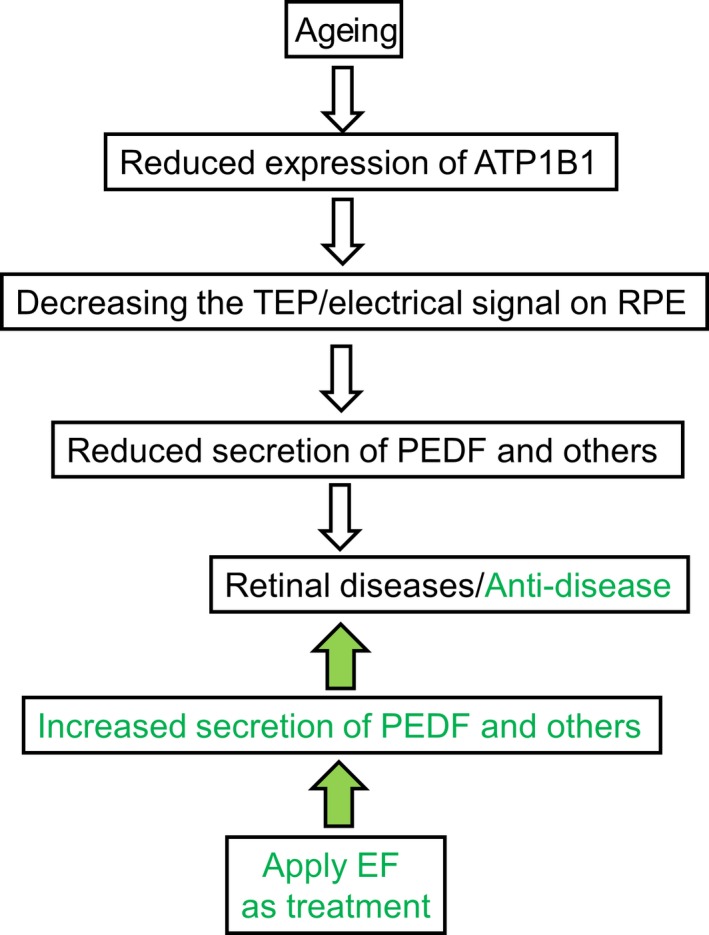
ATP1B1/TEP in AMD formation and possible therapeutic role. In ageing people (white arrows moving downward), a reduced TEP mediated by reduced ATP1B1 leads to decreased secretion of PEDF, which is an important factor in generation of AMD (white arrows indicate reductions and AMD onset). An applied EF offsets the reduced TEP and restores it to normal. A restored TEP increases the secretion of PEDF from RPE and may prevent the onset and epithelial degeneration associated with AMD (green arrows show boost to PEDF levels and improvement in course of AMD)

## 4 DISCUSSION

People with AMD lose their central vision, severely impairing their ability to read, watch television or drive. The epicentre of the disease is the retinal pigment epithelium (RPE), a single layer of cells in the retina adjacent to the photoreceptor cells. Dysfunction and death of RPE cells play a critical role in the pathogenesis of AMD. Pigment epithelium‐derived factor (PEDF) is a 50 kDa naturally occurring glycoprotein, a member of the serpin superfamily that is secreted by RPE cells from their apical membranes.^16^, ^17^, ^18^ PEDF acts as a neurotrophic factor and has neuroprotective properties. RPE cells secrete pigment epithelium‐derived factor (PEDF) into the interphotoreceptor matrix of the retina,^50^, ^51^ but the regulation of PEDF secretion is poorly understood. Here, we found that the endogenous TEP (the electrical potential difference across the RPE) regulates secretion of PEDF and may maintain the level of PEDF apically as the RPE ages.

### 4.1 Reduced ATP1B1 and TJ proteins cause a lower TEP

The Na^+^/K^+^‐ATPase is composed of three major polypeptides, α, β and γ‐subunit.^52^, ^53^ The α‐subunit is a multi‐span membrane protein with a molecular mass of 112 000 Da and is responsible for the catalytic and ion transport properties.^54^, ^55^ The β‐subunit is a transmembrane polypeptide with a molecular weight between 40 000 and 60 000 Da. The β‐subunit is essential for the normal activity of the enzyme and may facilitate the processing and insertion of the α‐subunit into the plasma membrane.^55^, ^56^, ^57^ Wetzel et al indicate that the subunits of sodium pumps in the different apical/basal faces imply that the Na^+^/K^+^‐ATPase has distinct physiological functions in the epithelium and that its activity is likely to be regulated by different mechanisms, for example sodium gradient formation for osmotic gradient and water diffusion.^58^ The Na^+^/K^+^‐ATPase (sodium pump) allows Na^+^ to accumulate in the apical extracellular space of the RPE, because it pumps three Na^+^ from the cytoplasm electrogenically out into the extracellular fluid, in exchange for two K^+^ ions entering the cells. In addition, high‐resistance electrical “seals” which are dependent on specific proteins (eg, TJ protein ZO‐1 and adherent protein E‐cadherin) exist between neighbouring cells in the RPE and these greatly reduce the electrical conductivity (and increase the resistance) between the apical and basal extracellular spaces. The same basic elements of polarized channels, pumps and tight junctions are found in most other epithelia, for example skin, cornea, kidney and also establish a TEP in these tissues. Therefore, normally a high concentration of Na^+^ will accumulate at the apical side of RPE and form a trans‐RPE electrical potential difference with the apical side positive.^4^, ^59^ Here we found that the ATP1B1 in older and DKO mice RPE was lower (by ~50%) than in younger and wild‐type mice. Consequently, the TEP in older and DKO mice should be lower. Using transwell culture (the Ussing chamber technique), we confirmed a much reduced TEP in DKO mice. These data are consistent with a conclusion that the reduced expressions of both ATP1B1 and of cell‐cell tight junction proteins underpin the age‐related decline in both TEP and TEER. Because the TEP plays functional roles in cell migration, division, polarization and development, we speculated that a reduced RPE TEP may play a role in age‐related retinal dysfunction diseases.

### 4.2 A blunted TEP reduced the secretion of PEDF in AMD

Pigment epithelial derived factor is a potent neurotrophic, anti‐inflammatory and anti‐senescence glycoprotein that protects the retinal neurons and photoreceptors against apoptosis during retinal degeneration and light‐induced retinal damage,^60^, ^61^, ^62^, ^63^ and its down‐regulation is linked to senescence in cultured fibroblast cells.^64^ Polarized hES‐RPE (human embryonic stem cell‐derived RPE) and polarized fRPE (foetal RPE) secreted PEDF at mg/ml levels; in contrast, non‐polarized hES‐RPE or fRPE and ARPE19 cells (a human retinal pigment epithelial cell line with differentiated properties) secreted PEDF at levels approximately 100‐fold less (*P* < 0.001).^65^ In addition, PEDF is one of the more potent antiangiogenic factors with demonstrable inhibitory activity against ocular neovascularization in vivo^22^ and a reduced PEDF expression in DKO mice contributes to retinal degeneration.^35^ In transwell culture, our data showed that the inhibition of ATPase and so the TEP with ouabain significantly reduced the secretion of PEDF from RPE cells.

Applied electric fields (EFs) that mimic the endogenous TEP regulate the behaviour of a variety of cells by determining their orientation, proliferation, differentiation, migration, polarization and expression of growth factors.^11^, ^66^, ^67^ Electric stimulation triggers NGF (nerve growth factor) production and secretion by electrically induced protein kinase C (PKC) activation.^68^ RPE cells secrete pigment epithelium‐derived factor (PEDF) into the interphotoreceptor matrix of the retina,^50^, ^51^ but the regulation of PEDF secretion is poorly understood. Using an applied EF to mimic the TEP, we found that the expression of PEDF and secretion of PEDF significantly increased in RPE cells. In addition, in RPE from DKO mice with deficient expression of ATP1B1, an applied EF still increased the expression of PEDF, suggesting that the applied EF regulated the expression of PEDF directly. These data further indicate that the TEP and PEDF release may be linked mechanistically and play a role in retinal degenerative disease (Figure 7).

### 4.3 Applying an EF to treat retinal disease

We have shown that applying an EF, to mimic the physiological TEP, could be of therapeutic use in retinal disease through regulation of secretion of PEDF by the RPE (Figure 7). However, the high complexity of structure and function in the eye is an obstacle to applying EFs in vivo. Recently, Ho et al have developed a wireless method to transfer electrical power deep into tissues. Their microimplant is 2 mm long, weighs 70 mg and can be transplanted into the chest to control the heart.^69^ This technology may be modifiable to supply an EF to treat retinal degeneration in vivo. In addition, the TEP can be amplified by specifically targeted drugs, such as aminophylline, AgNO_3_, PGE_2_ (prostaglandin E) and in corneal epithelium a drug‐amplified EF enhanced directed nerve and epithelial cell growth and promoted faster wound healing.^70^ Therefore, chemical regulators of the TEP may provide another clinical treatment for retinal diseases, perhaps in combination with an applied EF.

Electrical treatments such as heart pacemakers, deep brain stimulators and cochlear implants continue to have major clinical success. Similar treatments for retinal regeneration will only succeed if effective ways of harnessing and delivering an applied EF are identified. Our work paves the way for this and opens up new electrical therapeutic possibilities with significant clinical potential.

## AUTHOR CONTRIBUTIONS

LC, JL, JP, NL and CDM designed the experiments and analysed the data. LC, JL, JP, GM and AS performed the experiments. LC, JP, CDM, JVF and NL wrote and revised the manuscript. MC and HX supplied CCL2^−/−^/CX3CR1^−/−^ mice. All the authors reviewed the manuscript.

## COMPETING FINANCIAL INTERESTS

The authors declare no competing financial interests.
